# Prevalence and factors associated with chronic kidney disease and end-stage renal disease in HIV-1-infected Asian patients in Tokyo

**DOI:** 10.1038/s41598-017-15214-x

**Published:** 2017-11-06

**Authors:** Takeshi Nishijima, Yohei Kawasaki, Yoshikazu Mutoh, Kiyomi Tomonari, Kunihisa Tsukada, Yoshimi Kikuchi, Hiroyuki Gatanaga, Shinichi Oka

**Affiliations:** 1 AIDS Clinical Center, National Center for Global Health and Medicine, Tokyo, Japan; 2 Department of Drug Evaluation & Informatics, Graduate School of Pharmaceutical Sciences, University of Shizuoka, Shizuoka, Japan

## Abstract

This single-center cross-sectional study determined the prevalence and factors associated with chronic kidney disease (CKD) and end-stage renal disease (ESRD) in HIV-1-infected Asian patients at the largest HIV clinic in Japan. HIV-1-infected patients who visited the clinic between September and December 2016 were analyzed. CKD was defined as estimated glomerular filtration rate of <60 ml/min/1.73 m^2^ or proteinuria ≥1+, observed at least over three months. A logistic regression model was used to estimate the effects of various variables on CKD. The study included 1,990 patients; with 97% Asians, 34% aged of ≥50 years, and 94% had HIV-1 load <50 copies/ml. The median time from HIV-1 diagnosis to study enrollment and duration of ART were 9.1 years (IQR4.8–14.2) and 7.35 years (IQR3.28–12), respectively. CKD and ESRD were diagnosed in 256 (13%) and 9 (0.5%) patients, respectively. The prevalence of CKD was 18.6% for age 50–59, 28.5% for 60–69, and 47% for over 70. Older age, heavier body weight, diabetes mellitus, hypertension, and longer duration of ART, but not duration of TDF exposure, were associated with CKD. The traditional risk factors, rather than HIV-1-related variables, were associated with CKD, suggesting the importance of management of such comorbidities in maintenance of renal function.

## Introduction

The advent of antiretroviral therapy (ART) substantially improved the prognosis of patients with HIV-1 infection^1^. With increased age of HIV-1-infected patients, the importance of management of non-communicable diseases (NCDs) cannot be overemphasized^2^. Chronic kidney disease (CKD) and end-stage renal disease (ESRD) are one of the important NCDs that influence morbidity and mortality^3,4^. Maintenance of renal function is particularly important for HIV-1-infected patients, since HIV-1 infection is currently not curable and a life-long treatment is required. Furthermore, the rate of deterioration of renal function is reported to be faster in HIV-1-infected patients than the general population^5,6^.

Few studies in Asia have assessed the prevalence and factors associated with CKD and ESRD in patients with HIV-1 infection^7–9^. HIV-1-infected Asians might be more susceptible to dose-dependent nephrotoxic medications, such as tenofovir disoproxil fumarate (TDF), due to their small body weight in general, compared to Whites or Blacks, as confirmed by our previous studies as well as studies from India, Thailand, and Vietnam^10–13^. HIV-1-infected Asians are living longer, especially in resource-rich setting, and this further highlights the importance of maintenance of renal function in such patients.

The aim of the present study was to determine the prevalence and factors associated with CKD and ESRD in HIV-1-infected patients in Asia, with a particular focus on TDF nephrotoxicity, and to define the clinical characteristics of HIV-1-infected patients with ESRD.

## Methods

### Study design and patients

We performed a single-center cross-sectional study of HIV-1-infected patients at the AIDS Clinical Center, National Center for Global Health and Medicine (NCGM), Tokyo. The inclusion criteria were *1)* HIV-1-infected patients who visited the clinic between September to December 2016; *2)* available serum creatinine and CD4 count data during the study period. The following exclusion criteria were applied: *1)* Patients aged <20; *2)* patients hospitalized throughout the study period.

The study was approved by the Human Research Ethics Committee of NCGM (G-002168-00). Informed consent was waived because this study only used data gained from routine clinical practice. However, the study patients included 943 patients who participated in our kidney tubular study, which systematically examined urine protein and tubular markers during the same period, and written informed consent was obtained from these patients. The study was conducted according to the principles expressed in the Declaration of Helsinki.

### Measurements

eGFR was calculated using the following Japanese equation based on standardized serum creatinine, sex, and age, developed by the Japanese Society of Nephrology: eGFR = 194 × [serum creatinine]^−1.094^ × [age]^−0.287^ × [0.739 for females]^14^. This equation was chosen because it performs better than The Chronic Kidney Disease Epidemiology Collaboration (CKD-EPI) equation^15^ in patients with small body stature, such as Japanese^13,16,17^.

All potential factors associated with CKD, with special focus on the duration of TDF exposure, were recorded, together with basic demographic data, from the medical records^18–21^. These included duration of TDF exposure, history of TDF use, current TDF use, time from the diagnosis of HIV-1 infection, duration of ART, current ART regimen, age, sex, height, body weight, body mass index (BMI; body weight [kg]/height [m]^2^), history of AIDS (defined as history of or concurrent presence of one of the 23 AIDS-defining diseases including opportunistic infections and malignancies set by the Japanese Ministry of Health, Labour and Welfare)^22^, HIV-1 transmission route, laboratory data (CD4 cell count, HIV viral load, and serum creatinine level, urine dipstick test), and presence or absence of other medical conditions (concurrent nephrotoxic drugs, such as ganciclovir, NSAIDs, and sulfamethoxazole/trimethoprim; diabetes mellitus defined as the use of glucose-lowering agents; hypertension defined as current treatment with antihypertensive agents; dyslipidemia defined as current treatment with lipid-lowering agents; hepatitis B infection defined as positive hepatitis B surface antigen; hepatitis C infection defined as positive HCV antibody; and smoking (if one ever smoked or not). Patients visited our clinic at least every 3 months for measurement of CD4 cell count, HIV-1 viral load, and eGFR, since the prescription period under the Japanese health care system is limited to 3 months^23^. The data of the latest day during the study period when both serum creatinine and CD4 counts were measured were used. Furthermore, for 943 patients who participated in our kidney tubular function study, urine albumin values were collected from the medical records.

### Statistical analysis

CKD was defined as eGFR <60 ml/min/1.73 m^2^ or proteinuria by urine dipstick test of ≥1+, each observed for at least three months^9,24^. Furthermore, patients were divided into four groups according to the eGFR values observed for at least three months: patients with eGFR of <15, 15–29, 30–44, and 45–59 ml/min/1.73 m^2^. In addition, patients on chronic renal dialysis were also identified. Due to shortage of donors, it is common to introduce dialysis for patients with ESRD in Japan, instead of kidney transplantation^25^. A logistic regression model was used to estimate the effect of duration of TDF exposure and other variables on CKD. Variables with *p* value less than 0.05 were incorporated into the multivariate model. Time from diagnosis of HIV-1 infection was not incorporated into the multivariate model because of multicollinearity with duration of ART. Patients with available urine albumin values (n = 943) were categorized for risk of CKD progression according to the KDIGO classification based on eGFR and severity of albuminuria^26^.

Statistical significance was defined with 2-sided *p* values of <0.05. We used odds ratios (ORs) with 95% confidence intervals (95% CIs). All statistical analyses were performed with SAS software, version 9.4 (SAS Institute, Cary, NC).

## Results

**Figure 1:**
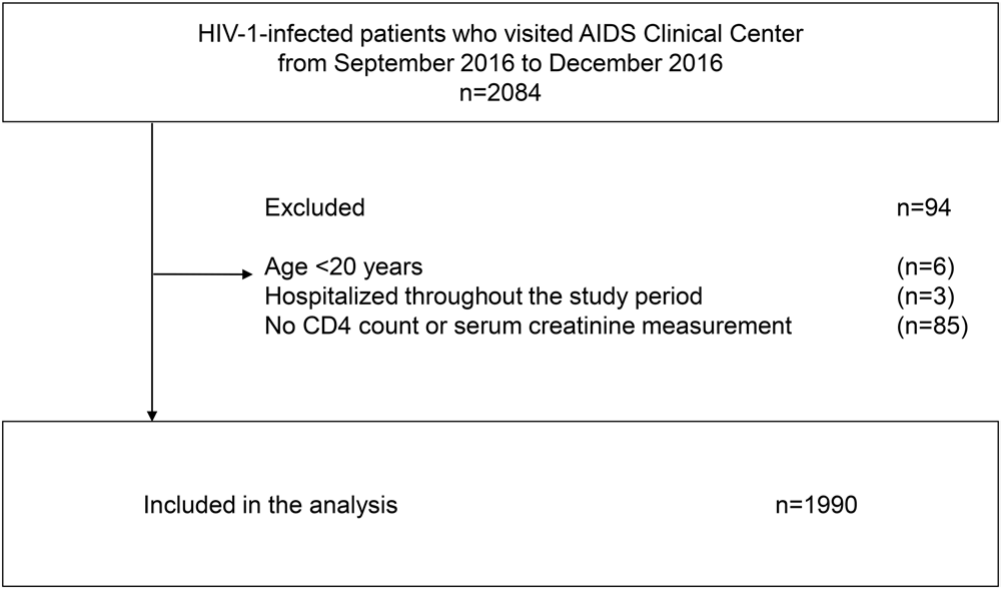
Study population.

Of the 2,084 patients with HIV-1 infection who visited our clinic during the study period, 94 patients were excluded and data of the 1,990 study patients were analyzed (Fig. 1). Of these, 97% were Asians and 92% were Japanese. 1,828 (92%) were males with a median age of 45 (IQR 39–53) (Table 1). With regard to age, 34% of patients were older than 50 years and 14% were older than 60 years. The median body weight was 66.7 kg (IQR 59.3–75.2) and median BMI was 23.2 kg/m^2^ (IQR 21.2–25.7). Furthermore, 1,587 (80%) patients self-identified as men who have sex with men. The HIV-1 viral load was <50 copies/ml in 1,861 (94%) while only 51 (3%) patients were treatment-naïve for HIV-1 infection. 1,819 (91%) patients were taking standard ART with one key drug [either protease inhibitor, integrase inhibitor, or non-nucleotide reverse transcriptase inhibitor (non-NRTI)] plus two NRTIs. The median time from diagnosis of HIV-1 infection to study enrolment was 9.1 years (IQR 4.8–14.2), and median duration of ART was 7.35 years (IQR 3.28–12).

**Table 1:** Characteristics of the study patients, patients with and without chronic kidney disease.

Baseline characteristics	All patients (n = 1990)	CKD group (n = 256)	Non-CKD group (n = 1734)	p value
Sex (male), n (%)	1828 (92)	245 (95)	1583 (92)	
Age^†^	45 (39–53)	54 (46–65)	44 (38–51)	^#^
History of TDF use, n (%)	1216 (61)	156 (60)	1060 (61)	
Current TDF use, n (%)	774 (39)	69 (27)	705 (41)	^#^
TDF duration (years)^†^	1.82 (0–5.34)	1.28 (0–5.43)	1.92 (0–5.32)	
TDF duration for patients with history of TDF use (years)^†^	n = 1216, 4.44 (2.37–6.91)	n = 156, 4.28 (1.98–6.99)	n = 1060, 4.48 (2.42–6.89)	
Weight (kg)^†^	66.7 (59.3–75.2)	68 (62.5–78)	66 (59–75)	^*^
BMI (kg/m^2^)^†^	23.2 (21.2–25.7)	23.9 (22.1–26.2)	23.1 (21–25.6)	^#^
Race, n (%)				
Japanese	1823 (92)	244 (94)	1579 (91)	^*^
Asians other than Japanese	112 (6)	6 (2)	106 (6)	
Others	55 (3)	9 (4)	46 (3)	
eGFR (ml/min per 1.73 m^2^)^†^	74.9 (64.7–86.2)	53.3 (47.6–57.6)	77.1 (68.4–87.6)	^#^
Serum creatinine (mg/dl)^†^	0.87 (0.76–0.98)	1.14 (1.04–1.27)	0.85 (0.75–0.94)	^#^
CD4 cell count (/μl)^†^	561 (420–731)	554 (416–717)	563 (420–733)	
HIV RNA viral load < 50 copies/ml, n (%)	1861 (94)	253 (98)	1608 (93)	^#^
Route of transmission, n (%)				
Homosexual contact	1569 (79)	193 (75)	1376 (80)	
Heterosexual contact	295 (15)	46 (18)	249 (14)	
Injection drug use	24 (1)	3 (1)	21 (1)	
Contaminated blood product	80 (4)	15 (6)	65 (4)	
Unknown/others	22 (1)	2 (0.8)	20 (1)	
Smoking, n (%)	881 (44)	77 (30)	804 (46)	^#^
Hypertension, n (%)	284 (14)	95 (37)	189 (11)	^#^
Diabetes mellitus, n (%)	102 (5)	37 (14)	65 (4)	^#^
Dyslipidaemia, n (%)	349 (18)	76 (29)	273 (16)	^#^
Current use of nephrotoxic drugs, n (%)	202 (10)	19 (7)	183 (11)	
Hepatitis B virus infection, n (%)	180 (9)	23 (9)	157 (9)	
Hepatitis C virus infection, n (%)	186 (9)	28 (11)	158 (9)	
History of AIDS, n (%)	583 (29)	98 (38)	485 (28)	^#^
ART regimen, n (%)				
2NRTIs + key drug	1819 (91)	206 (80)	1613 (93)	^#^
NRTI spare	89 (5)	41 (16)	48 (3)	^#^
PI/r, n (%)	570 (29)	70 (27)	500 (29)	
Atazanavir/ritonavir	62 (3)	3 (1)	59 (3)	
Darunavir/ritonavir	403 (20)	52 (20)	351 (20)	
Lopinavir/ritonavir	67 (3)	7 (3)	60 (4)	
Fosamprenavir/ritonavir	39 (2)	8 (3)	31 (2)	
NNRTI, n (%)	234 (12)	48 (19)	186 (11)	^#^
Nevirapine	22 (1)	5 (2)	17 (1)	
Efavirenz	65 (3)	8 (3)	57 (3)	
Etravirine	25 (1)	13 (5)	12 (0.7)	^#^
Rilpivirine	122 (6)	22 (9)	100 (6)	
INSTI, n (%)	1219 (61)	189 (73)	1030 (60)	^#^
Raltegravir	232 (12)	52 (20)	180 (10)	^#^
Dolutegravir	794 (40)	126 (49)	668 (39)	^#^
E/C/F/TAF	194 (10)	11 (4)	183 (11)	^#^
E/C/F/TDF	1 (0.1)	0	1 (0.1)	
PI, n (%)	21 (1.1)	3 (1)	18 (1)	
Atazanavir	5 (0.3)	1 (0.4)	4 (0.2)	
Nelfinavir	5 (0.3)	0	5 (0.3)	
Fosamprenavir	11 (0.6)	2 (0.8)	9 (0.5)	
Abacavir	874 (44)	135 (52)	739 (43)	^*^
Maraviroc	3 (0.2)	1 (0.4)	2 (0.1)	
Zidovudine	3 (0.2)	0	3 (0.2)	
Treatment-naive, n (%)	51 (3)	1 (0.4)	50 (3)	^*^
Time from diagnosis of HIV-1 infection (years)^†^	9.10 (4.80–14.2)	10.6 (7.2–16.3)	8.90 (4.5–13.8)	^#^
ART duration (years)^†^	7.35 (3.28–12)	9.12 (5.90–15.0)	6.88 (3.08–11.5)	^#^

^†^(median, interquartile range), *p value < 0.05 and ≥0.005, ^#^p value < 0.005, CKD: chronic kidney disease, TDF: tenofovir disoproxil fumarate, BMI: body mass index, eGFR: estimated glomerular filtration rate, ART: antiretroviral therapy, NRTI: nucleotide reverse transcriptase inhibitor, PI/r: ritonavir-boosted protease inhibitor, NNRTI: non-nucleotide reverse transcriptase inhibitor, INSTI: integrase strand transfer inhibitor, E/C/F/TAF: elvitegravir/cobicistat/emtricitabine/tenofovir alafenamide, E/C/F/TDF: elvitegravir/cobicistat/emtricitabine/tenofovir disoproxil fumarate.

**Figure 2:**
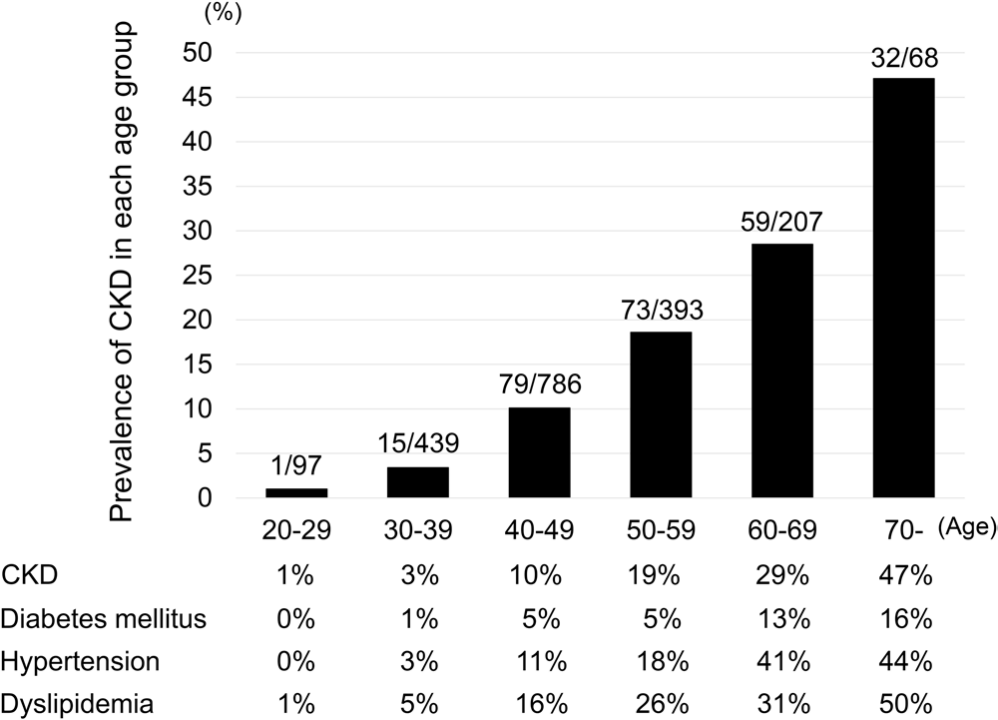
Prevalence of CKD and other comorbidities in each age group. CKD: chronic kidney disease.

The protocol-defined CKD was identified in 256 (13%) patients [the results of urine dipstick test were missing in 345 (17%) patients, including 31 patients who were identified as CKD based on eGFR value]. Patients with CKD were significantly older, had heavier body weight and higher BMI, were more likely to have history of AIDS, hypertension, diabetes mellitus, dyslipidemia, and were less likely to smoke than those without CKD (Table 1). Also, the time from HIV diagnosis and duration of ART were longer in patients with CKD than those without CKD. More patients with CKD were taking NRTI sparing regimen of ART than those without CKD, and a standard regimen of one key drug plus two NRTIs was more common among patients without CKD. Figure 2 shows the prevalence of CKD in each age group. The prevalence of CKD increased with increased age: 1% for patients aged 20–29, 3.4% 30–39, 10.1% for 40–49, 18.6% for 50–59, 28.5% for 60–69, and 47% for patients aged >70.

Univariate analysis showed that duration of TDF exposure was not associated with CKD (per 1 year increase, OR 1.00, 95% CI 0.96–1.04) (Table 2). Older age (per 1 year increase, OR 1.08, 95% CI 1.07–1.09), heavier body weight (per 1 kg increase, OR 1.01, 95% CI 1.00–1.02), diabetes mellitus (OR 4.27, 95% CI 2.79–6.55,), hypertension (OR 4.73, 95% CI 3.52–6.34), dyslipidemia (OR 2.22, 95% CI 1.65–2.99), history of AIDS (OR 1.56, 95% CI 1.19–2.05), longer duration of ART (per 1 year increase, OR 1.07, 95% CI 1.05–1.09), and longer time from diagnosis of HIV-1 infection (OR 1.04, 95% CI 1.02–1.06) were significantly associated with CKD. Smoking was inversely associated with CKD (OR 0.49, 95% CI 0.37–0.65).

**Table 2:** Results of uni- and multi-variate analyses for the association of various factors with chronic kidney disease.

	OR	95% CI	p value	Adjusted OR	95% CI	p value
Duration of TDF exposure (per 1 year increase)	1.00	0.96–1.04	0.835			
Age (per 1 year increase)^†^	1.08	1.07–1.09	<0.001	1.07	1.05–1.08	<0.001
Male sex	1.64	0.93–2.88	0.087			
Body weight (per 1 kg increase)^†^	1.01	1.00–1.02	0.005	1.02	1.01–1.04	<0.001
Current CD4 count (per 1/µl increase)	1.00	1.00–1.00	0.66			
History of AIDS^†^	1.56	1.19–2.05	0.001	1.27	0.94–1.71	0.12
Use of nephrotoxic drugs	0.67	0.41–1.10	0.11			
Dyslipidemia^†^	2.22	1.65–2.99	<0.001	0.97	0.69–1.36	0.85
Diabetes mellitus^†^	4.27	2.79–6.55	<0.001	1.96	1.22–3.16	0.006
Hypertension^†^	4.73	3.52–6.34	<0.001	1.97	1.40–2.77	<0.001
Smoking^†^	0.49	0.37–0.65	<0.001	0.60	0.44–0.82	0.001
Positive hepatitis B antigen	0.98	0.62–1.55	0.92			
Positive HCV antibody	1.21	0.79–1.85	0.39			
Duration of ART (per 1 year increase)^†^	1.07	1.05–1.09	<0.001	1.03	1.00–1.05	0.052
Time from diagnosis of HIV-1 infection (per 1 year increase)	1.04	1.02–1.06	<0.001			

^†^variables incorporated into the multivariate model.

TDF: tenofovir disoproxil fumarate, ART: antiretroviral therapy.

Multivariate model showed that older age (per 1 year increase, OR 1.07, 95% CI 1.05–1.08), heavier body weight (per 1 kg increase, OR 1.02, 95% CI 1.01–1.04), diabetes mellitus (OR 1.96, 95% CI 1.22–3.16), and hypertension (OR 1.97, 95% CI 1.40–2.77) persisted to be significantly associated with CKD, while longer duration of ART was marginally associated with CKD (per 1 year increase, OR 1.03, 95% CI 1.00–1.05, p = 0.052). Smoking was inversely associated with CKD (OR 0.60, 95% CI 0.44–0.82).

With regard to the eGFR categorization, 9 (0.5%) patients had eGFR of <15 ml/min/1.73 m^2^, 2 (0.1%) with eGFR of 15–29 ml/min/1.73 m^2^, 25 (1%) with eGFR of 30–44 ml/min/1.73 m^2^, and 181 (9%) patients with GFR of 45–59 ml/min/1.73 m^2^. The clinical characteristics of the 9 patients with ESRD (eGFR < 15 ml/min/1.73 m^2^) are shown in Table 3. All 9 patients were Japanese, 8 were on chronic hemodialysis and the other was scheduled for introduction to dialysis. The cause of ESRD was IgA nephropathy in 3 patients, diabetic nephropathy in 2, unknown in 2 patients, and membranoproliferative glomerulonephritis and acute renal failure associated with renal abscess in one patient each. All except one patient were taking NRTI-sparing regimen of ART. ESRD developed long after infection with HIV-1 in 5 patients, 2 patients became infected with HIV-1 after the commencement of dialysis, and HIV-1 was diagnosed in the other two patients on routine screening before surgery for arteriovenous fistula.

**Table 3:** Characteristics of patients with end-stage renal disease.

No.	Age (yrs)	Sex	Cause of ESRD	Route of HIV-1 infection	CD4 count (/μl)	HIV-1 viral load (copies/ml)	ART	Time from HIV-1 diagnosis (years)	Time from initiation of dialysis (years)
1	69	M	Unknown/HCV infection	Contaminated blood product	411	<50	DRV/r + RAL	33.4	6.8
2	67	F	MPGN/HCV infection	Contaminated blood product	917	<50	ETR + RAL	20.8	0.8
3	61	M	IgA nephropathy	Heterosexual contact	140	<50	DRV/r + RAL	8.4	19.4
4	48	M	Acute renal failure due to renal abscess	Heterosexual contact	428	<50	DRV/r + RAL	16.3	1.9
5	45	M	Diabetic nephropathy	Homosexual contact	428	<50	ETR + RAL	3.3	3.2
6	43	M	IgA nephropathy/HBV infection	Homosexual contact	314	<50	FPV + TDF	19.1	19.5
7	41	M	IgA nephropathy	Homosexual contact	450	<50	DTG + RPV	0.4	12.5
8	38	M	Diabetic nephropathy/HCV infection	Contaminated blood product	157	<50	ETR + RAL	33.5	1.8
9	43	M	Unknown	Homosexual contact	1684	<50	DRV/r + RAL	14.3	scheduled for dialysis

ESRD: end stage renal disease, M: male, F: female, MPGN: membranoproliferative glomerulonephritis, ART: antiretroviral therapy, DRV/r: ritonavir-boosted darunavir, RAL: raltegravir, ETR: etravirine, FPV: fosamprenavir, TDF: tenofovir disoproxil fumarate, DTG: dolutegravir, RPV: rilpivirine.

**Figure 3:**
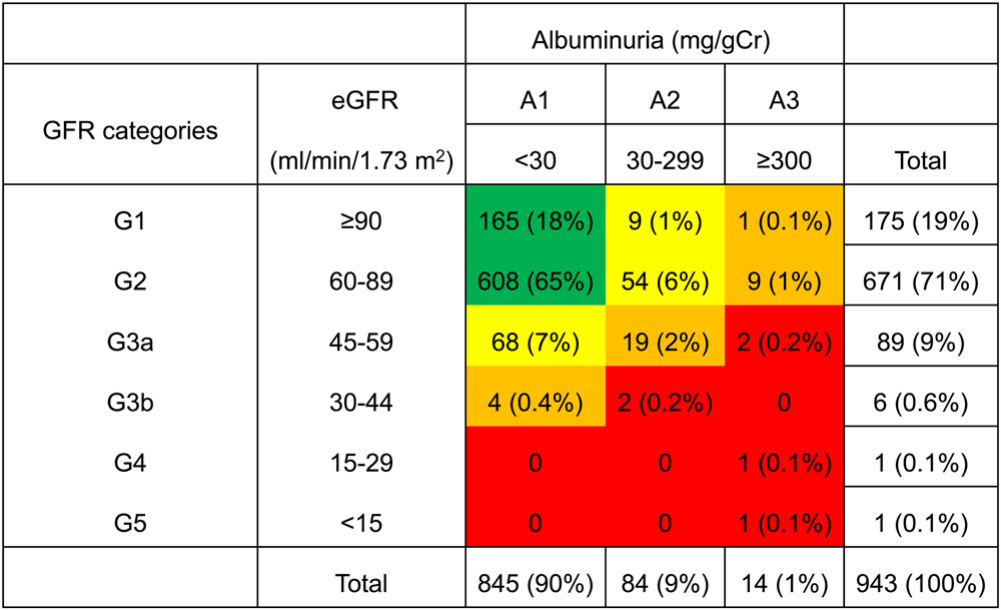
Risk categories of CKD according to 2012 KDIGO classification in subgroup of 943 patients. Patients were categorized into each risk category based on eGFR value and severity of albuminuria. Green: low risk; Yellow: moderately increased risk; Orange: high risk; Red, very high risk. CKD: chronic kidney disease, KDIGO: kidney disease: outcomes quality initiative, eGFR: estimated glomerular filtration rate.

Figure 3 lists the risk categories of CKD according to the 2012 KDIGO classification for 943 patients. Whereas 773 (82%) patients were categorized as the low risk group (green), the 170 (18%) patients of the other group [including 131 (14%) patients with moderately increased risk (yellow), 33 (3%) with high risk (orange), and 6 (0.6%) with very high risk (red)], were categorized to be at risk of CKD progression.

## Discussion

In this largest HIV clinic in Japan where 34% of patients were more than 50 years of age, the prevalence of CKD in HIV-1-infected patients was 13%. Older age, heavier body weight, diabetes mellitus, hypertension, and longer duration of ART were associated with CKD, whereas the duration of TDF exposure was not associated with CKD. The prevalence of CKD increased in the elderly: it was 29% in patients aged 60–69 and rose to 47% in patients aged >70. Only 9 (0.5%) patients were classified as ESRD, 8 of them on chronic dialysis. These results demonstrated the longevity of HIV-1-infected patients in resource-rich setting in Asia and also the high prevalence of CKD, especially among the elderly. The traditional risk factors, such as diabetes mellitus and hypertension, rather than HIV-1-related variables, were associated with CKD, suggesting the importance of management of such comorbidities in maintenance of renal function.

The present study has three important strengths. First, to our knowledge, this is the largest study that elucidated the prevalence and factors associated with CKD in HIV-1-infected patients in Asia. A few such studies are available at present; one from China (n = 322, CKD 16.8%)^7^, Japan (n = 1447, CKD 14.1%)^27^, and Taiwan (n = 512, CKD 7%)^8^. Since older age is an established risk for CKD^24^, the age distribution of the study patients can substantially affect the prevalence of CKD, and thus large studies are probably more appropriate to determine the true prevalence of CKD. In the present study, 34% of patients were more than 50 years of age, and the prevalence of CKD increased with age, with the peak value in elderly patients aged more than 70 years (Fig. 2). Also noteworthy was the high prevalence of comorbidities in the elderly, such as diabetes mellitus, hypertension, and dyslipidemia (Fig. 2).

Second, to our knowledge, this is also the first study that classified HIV-1 infected patients in Asia according to the 2012 KDIGO classification for risk of CKD progression based on eGFR and albuminuria (Figure 3)^26^. Because 2012 KDIGO classification was performed for the subgroup of 943 patients, we cannot directly compare the prevalence of CKD (either eGFR < 60 ml/min/1.73 m^2^ or proteinuria) in the whole study patients (13%, n = 1,990) and prevalence of risk categories according to the 2012 KDIGO classification. However, the prevalence of CKD among the subgroup (12.5%, n = 943) was lower than the prevalence of those categorized as either moderately increased risk, high risk, or very high risk among the same subgroup of patients (18%, n = 943) by the 2012 KDIGO classification. The 2012 KDIGO classification might screen a wider range of people at risk of CKD progression than the use of either CKD or non-CKD category.

Third, this study described in detail the clinical characteristics of patients with ESRD. It is noteworthy that of the 9 patients with ESRD, 5 developed ESRD long after the diagnosis of HIV-1 infection. The number of such patients is predicted to increase in the future with further improvement in the prognosis of HIV-1-infected patients. It is also notable that the age of ESRD patients varied from 30’s to 60’s, and IgA nephropathy and diabetic nephropathy, common causes for ESRD in Japan^28^, were also common among HIV-1-infected ESRD patients in this study. These data warrant strict adherence to the recommendation of the guidelines; monitoring of renal function based on serum creatinine at least twice a year and urinalysis or quantitative measure of albuminuria/proteinuria at least annually^24^. Furthermore, 3 out of 9 patients with ESRD were patients who were infected with HIV-1 through contaminated blood products, although there are only 80 such patients among the study patients. Such patients, mostly comprised of hemophiliacs infected with HIV-1 and HCV around 1983^29^, could be at higher risk of developing ESRD partly due to the long exposure to HIV-1 and concurrent HCV infection. The result of univariate analysis showing a significant association between time from the diagnosis of HIV-1 and CKD seem to support this hypothesis.

Assessment of the association between particular current ART regimen and CKD is difficult to conduct, because the attending physicians might change ART to less nephrotoxic ones for patients with impaired renal function or with risk factors for CKD. This can be particularly true for TDF, because in 2016 when the data for this study were collected, TDF nephrotoxicity was well-established and well-publicized. This is the reason we only incorporated the variable “duration of TDF exposure” into the logistic regression model. However, the results showed that the duration of TDF exposure was not associated with CKD, in contrast to some large studies, which showed positive association between TDF and CKD^5,30^. This is probably because our group has been aware of TDF nephrotoxicity^21,31–35^ and as a result, TDF could have been discontinued early for patients with CKD risk in this cohort.

TDF, one of the most widely used ART but is nephrotoxic, will be replaced with its prodrug tenofovir alafenamide. However, other antiretroviral drugs which inhibit excretion of creatinine in the renal proximal tubules and increase serum creatinine value, such as dolutegravir, cobicistat, rilpivirine, raltegravir, and ritonavir^36^, will be widely used in both resource rich and resource poor settings and might further complicate the interpretation of eGFR value calculated using serum creatinine.

We need to acknowledge several limitations in this study. First, due to the nature of single-center study, selection bias could not be completely avoided. However, as mentioned above, this study is the largest to date that elucidated the prevalence of CKD in HIV-1-infected patients in Asia. Second, although the criteria for CKD were either eGFR < 60 ml/min/1.73 m^2^ or persistent proteinuria, urine dipstick test was not conducted in 345 patients, and thus the true prevalence of CKD in this study might be underestimated. However, considering that for those with available urine data (n = 1,645), 1,459 had eGFR of >60 ml/min/1.73 m^2^ and of these, 95 (6.5%) were diagnosed with CKD based on proteinuria. If we apply these data to patients without data on proteinuria, 20 extra patients (6.5% of 314 patients without urine data and with eGFR > 60 ml/min/1.73 m^2^) would have been diagnosed with CKD. Adding these 20 patients would slightly increase the prevalence of CKD from 13% to 14%. Third, although the main focus of this study was on HIV-1-infected patients in Asia, most study subjects were Japanese men, and only a small number of females were included.

In conclusion, the prevalence of CKD and ESRD was 13% and 0.5%, respectively, in this large Asian cohort of HIV-1-infected patients where 34% were more than 50 years of age. The prevalence of CKD was higher in the elderly, and comorbidities, such as diabetes mellitus and hypertension, were associated with CKD. Although longer duration of ART was associated with CKD, the duration of TDF use was not. These findings emphasize the longevity of HIV-1-infected patients in resource-rich setting in Asia and stress the importance of management of traditional risk factors for CKD, such as diabetes mellitus and hypertension, in such patients.
